# Motion Planning and Iterative Learning Control of a Modular Soft Robotic Snake

**DOI:** 10.3389/frobt.2020.599242

**Published:** 2020-12-03

**Authors:** Ming Luo, Zhenyu Wan, Yinan Sun, Erik H. Skorina, Weijia Tao, Fuchen Chen, Lakshay Gopalka, Hao Yang, Cagdas D. Onal

**Affiliations:** ^1^School of Mechanical and Materials Engineering, Washington State University, Pullman, WA, United States; ^2^Robotics Engineering Department, Worcester Polytechnic Institute, Worcester, MA, United States; ^3^Robotics Engineering and Mechanical Engineering Departments, Worcester Polytechnic Institute, Worcester, MA, United States

**Keywords:** soft robotics, pneumatics, snake robot, motion planning, feedback control

## Abstract

Snake robotics is an important research topic with a wide range of applications, including inspection in confined spaces, search-and-rescue, and disaster response. Snake robots are well-suited to these applications because of their versatility and adaptability to unstructured and constrained environments. In this paper, we introduce a soft pneumatic robotic snake that can imitate the capabilities of biological snakes, its soft body can provide flexibility and adaptability to the environment. This paper combines soft mobile robot modeling, proprioceptive feedback control, and motion planning to pave the way for functional soft robotic snake autonomy. We propose a pressure-operated soft robotic snake with a high degree of modularity that makes use of customized embedded flexible curvature sensing. On this platform, we introduce the use of iterative learning control using feedback from the on-board curvature sensors to enable the snake to automatically correct its gait for superior locomotion. We also present a motion planning and trajectory tracking algorithm using an adaptive bounding box, which allows for efficient motion planning that still takes into account the kinematic state of the soft robotic snake. We test this algorithm experimentally, and demonstrate its performance in obstacle avoidance scenarios.

## 1. Introduction

Compared with traditional rigid robots, soft robots allow for inherently safe contact with and adaptation to the environment, which can benefit robots in fields including human interaction, search and rescue and medical (Kim et al., 2013; Lee et al., 2017). However, soft materials and actuation methods also impede the advancement of autonomous soft robotics for the two following reasons. First, there are limited internal sensing technologies capable of measuring the deformation of soft mobile robots. One example of work done in this area is an untethered soft mobile robot with impressive durability (Tolley et al., 2014). However, this robot is teleoperated with no on-board sensory feedback for autonomous control. An additional work included a soft 2D manipulator that is capable of inserting itself though narrow maze-like structures using motion planning and control algorithms (Marchese et al., 2016), but the large external actuation system and external sensors make this method unsuitable for the mobile robot systems. Second, traditional motion planning and control techniques often require precise kinematic models. Soft robots are very difficult to model because a continuously deformable body exhibits infinite degrees-of-freedom (DoF) and non-linear mechanical dynamic response, making traditional methods of dynamic modeling, motion planning, and control computationally expensive.

To demonstrate the potential of an autonomous soft mobile robot, we introduce our fourth generation WPI Soft Robotic Snake (SRS). This robot can self-correct its gait by utilizing internal on-board curvature sensing technology and can navigate through the objects-filling environment. These are requirements for inspection and search-and-rescue applications, where a robotic snake is a salient solution due to its ability to navigate unstructured terrain while still being able to pass through narrow openings and complex passages (Dowling, 1996; Hopkins et al., 2009; Bogue, 2014). Currently, many snake robots are developed for these hazardous and complex applications. For example, The ACM series of robots demonstrate an evolution in snake robotics from the 2D motion of the ACM III to 3D motion, waterproof and other advances of the ACM-R4 (Yamada and Hirose, 2006). The Anna Konda is a snake robot that moves using a sidewinding gait and mounts two nozzles on the “head” module to spray water to put out fires (Liljeback et al., 2006). Similarly, the Aiko is a portable DC motor-operated platform for experimenting with snake robot locomotion (Transeth et al., 2008). The PIKo is a snake-like robot for internal inspection of complex pipe structures. It has eight degrees of freedom (DoF) and can traverse both horizontal and vertical pipe structures (Fjerdingen et al., 2009). A modular snake robot which can operate inside steam pipes, vessels and other confined spaces was presented in McKenna et al. (2008). The AmphiBot I and II snake robots were inspired by snakes and elongated fish such as lampreys and represent a novel type of robot with dexterous locomotion abilities. These were used to investigate hypotheses of how central nervous systems execute these abilities in animals (Crespi and Ijspeert, 2006). Another kind of amphibious robotic snake with modularized joints was introduced in Yang et al. (2015) for amphibious inspection, etc. On a different direction, the propulsion of the OmniTread snake robots was achieved by tank treads on all four sides of every link, which can help the robot move in complex environments (Armada et al., 2005). More recently, the OSMOS snake utilizes spherical shaped modules to help realize gaits without changing the robot body shape and orientation (Singh et al., 2017). Meanwhile, a lot of researchers have made progress on the study of snake robot locomotion. An algorithm called conditioned basis array factorization was used in Gong et al. (2016) to project high-dimensional trajectory data to low-dimensional snake robot control which improves the agility and maneuverability of the robot. A decentralized control scheme was introduced in Kano et al. (2017), the snake robot can automatically adjust gaits with Tegotae, a system which help the robot to evaluate the similarity of its generated action and perceived action. In Astley (2020), the researchers show the increased multi-articular muscle span can improve maximum cantilever performance and metabolic savings by using mathemetical model, and the results are compatible with research on snake robots (Kano et al., 2011). Researchers are also studying locomotion and path planning in cluttered environment. In Singh et al. (2018), the authors map the contact forces with the a viscous friction model for the snake robot, and utilize the strategy to plan the simplest trajectory for the robot with sufficient contacts with the obstacles. In Wang et al. (2020), the snake robot reacts to unmodeled complex terrain situation with force sensing information obtained from their joints, and uses directional compliance control strategy to change the stiffness of the body to overcome obstacles.

In our previous work, we developed three generations of the WPI SRS. The first generation (2013) was the first soft robotic snake (Onal and Rus, 2013). The second generation WPI SRS (2015) is able to run at 220 mm/s, which is around one body length per second (about 10 times faster than the first generation), making it one of the fastest controllable soft mobile robots (Luo et al., 2015b). The third generation WPI SRS (2015) is a self-contained soft mobile robot with embedded flexible curvature sensors (Luo et al., 2015a), able to operate without an external pressure source. The flexible curvature sensors utilize a Hall-Effect chip and a miniature magnet mounted on a flexible substrate, converting changes in magnetic field to curvatures. Our experiments show that this sensor technique has a faster dynamic response and is more portable than commercial curvature sensors (Ozel et al., 2016). In addition, we proposed a dynamic model of the WPI SRS, combining general snake-like kinematic modeling approaches with a soft actuator dynamic model and experimentally verified our model accuracy. This work overcomes the complexity of soft robotic modeling and provides a foundation for our research in control and motion planning for soft robots (Luo et al., 2014).

**Figure 1:**
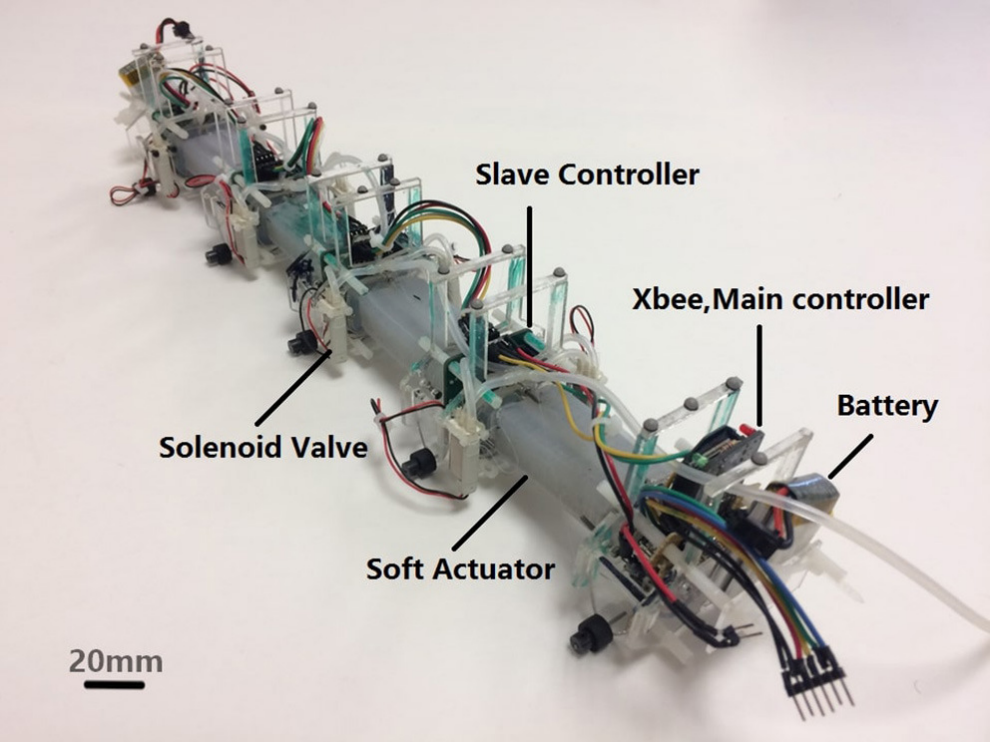
WPI SRS-4 is a modular autonomous soft robotic snake with embedded curvature sensing and local feedback controllers at each module.

**Figure 2:**
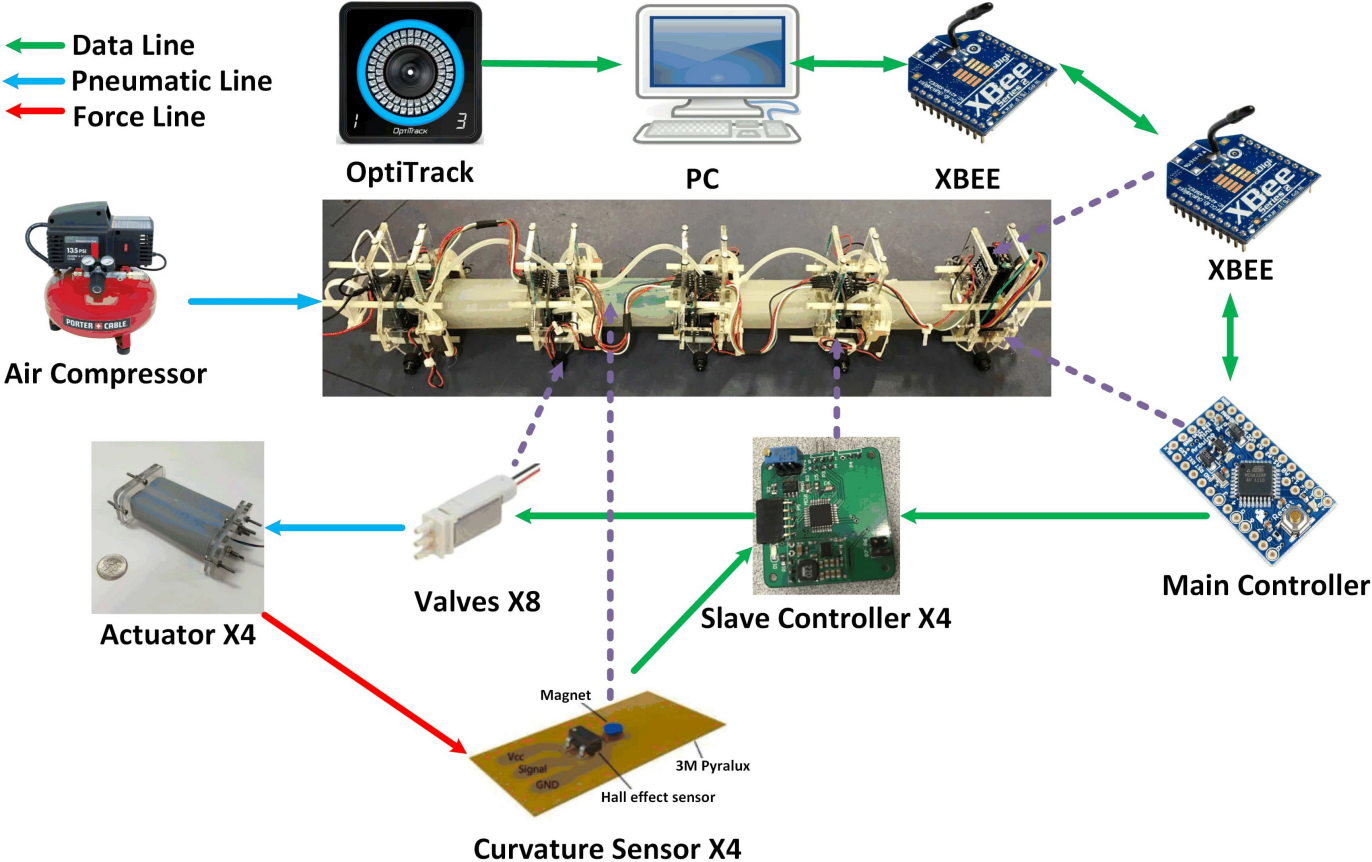
The System Architecture of WPI SRS-4.

The purpose of this paper is to advance the state of the art of soft snake robots by discussing a new soft snake with accompanying advances in locomotion consistency and motion planning. We present the WPI SRS-4, which is a highly integrated modular soft mobile robotic system. Each module is equipped with independent embedded soft curvature sensing, cascaded feedback control of on-board valves, and communications between modules (Figures 1, 2). Compared to the previous generations of soft snakes, this robot has the potential to perform additional functions with redundant modules (such as mobile manipulation) in the future, thanks to the scalable number of the modules. Each individual module is more durable due to advances in our fabrication techniques (Tao et al., 2015). In addition, we optimized the sensor design to increase performance for applicable curvature ranges applicable for serpentine locomotion (Luo et al., 2017; Skorina et al., 2017).

Due to the on-board curvature sensing and actuation, WPI SRS-4 is able to achieve autonomous navigation, which is the first for a soft mobile robot. We combine iterative learning control (Moore and Xu, 2000) with the on-board curvature sensors to enable the robot to correct its serpentine gait to follow consistent desired trajectories. In addition, we propose an “adaptive bounding box” method for motion planning of the SRS-4. This technique encapsulates the footprint of the snake robot within a dynamic area, allowing for efficient planning using motion primitives and a Bidirectional A* algorithm. The experimental result verify the usability of these two algorithms and present methods that enable the adaptation of existing algorithms for soft robotics.

The contributions of this work include:

- The first modular pressure-operated soft robotic snake with independent modules that integrate sensing, control, communication, and actuation subsystems.
- The first soft mobile robot self-correcting its gait using on-board sensors.
- The first motion planning and trajectory tracking control algorithms for a soft mobile robot.

## 2. WPI SRS System Architecture and Locomotion

The fourth generation WPI SRS has four soft bidirectional bending modules (made of two soft actuation chambers on both sides of an inextensible flexible constraint layer), each with its own local control board and on-board solenoid valves. In addition, SRS-4 has a single XBee for wireless communication, battery pack, and a separate main microcontroller, similar to our previous prototype (Luo et al., 2015a). The big difference on this generation is the lack of an on-board pressure source, which was deemed too heavy for the desired locomotion performance. Figure 2 shows the system architecture of SRS-4. Because this version is a modular system, each bending module has an independent slave controller which uses the local curvature sensor data of the bending module and control the two digital solenoid valves, which are used to alternately pressurize and vent each actuator chamber. The master controller, located at the tail of the snake, sends commands to the individual modules and receives bending angle data from the slave controllers using I2C serial communication. The pressure source is an external air compressor which we regulate down to 20 psi. We put infrared (IR) markers on the ends of each bending module, and used an external Optitrack motion capture system to calibrate each module's curvature sensor and detect the global pose and local state of the SRS-4. The PC can communicate wirelessly with the XBee sending control commands and receiving on-board sensor readings.

The gait we use for the SRS-4 is the serpentine locomotion, also called lateral undulation (Luo et al., 2017). Serpentine locomotion is executed by creating a traveling sinusoidal curvature wave down the length of the snake. Each module follows the same sinusoidal wave signal with a corresponding phase delay. The module will actuate in one direction if the sine wave is above an offset value and actuate in the opposite way otherwise. The control formulation for this type of gait is as follows:

(1) $$\begin{array}{l} S_{i}=u_{i}\text{sign}\left(\sin\left(\omega t+\beta_{i}\right)+\phi \right). \end{array}$$

In this equation, ω is the gait frequency, β_*i*_ is the traveling wave phase delay for the *i*th module. As, the snake shape has four modules and the full snake should form a complete sinusoid, these are usually set in increments of π/2. ϕ is the steering offset and will cause the snake to turn if non-zero Luo et al. (2017), while *S*_*i*_ modulates the behavior of the solenoid binary valves driving the *i*th module. The valve on one side of the bending module is opened at a pulse-width-modulation (PWM) duty cycle of *u*_*i*_, while the valve actuating the opposite side has the opposite duty cycle of (1 − *u*_*i*_). The algebraic sign of *S*_*i*_ defines the bending actuation direction, as we have previously discussed in Luo et al. (2015a). Here, *u*_*i*_ defines the control amplitude (duty cycle value of the solenoid valve). In our previous work, we set *u*_*i*_ = 1, meaning that our control was entirely bang-bang. In this paper, *u*_*i*_ varies in order to improve SRS performance, as discussed in section 4).

## 3. Locomotion Dynamics of a Modular Soft Robotic Snake

**Figure 3:**
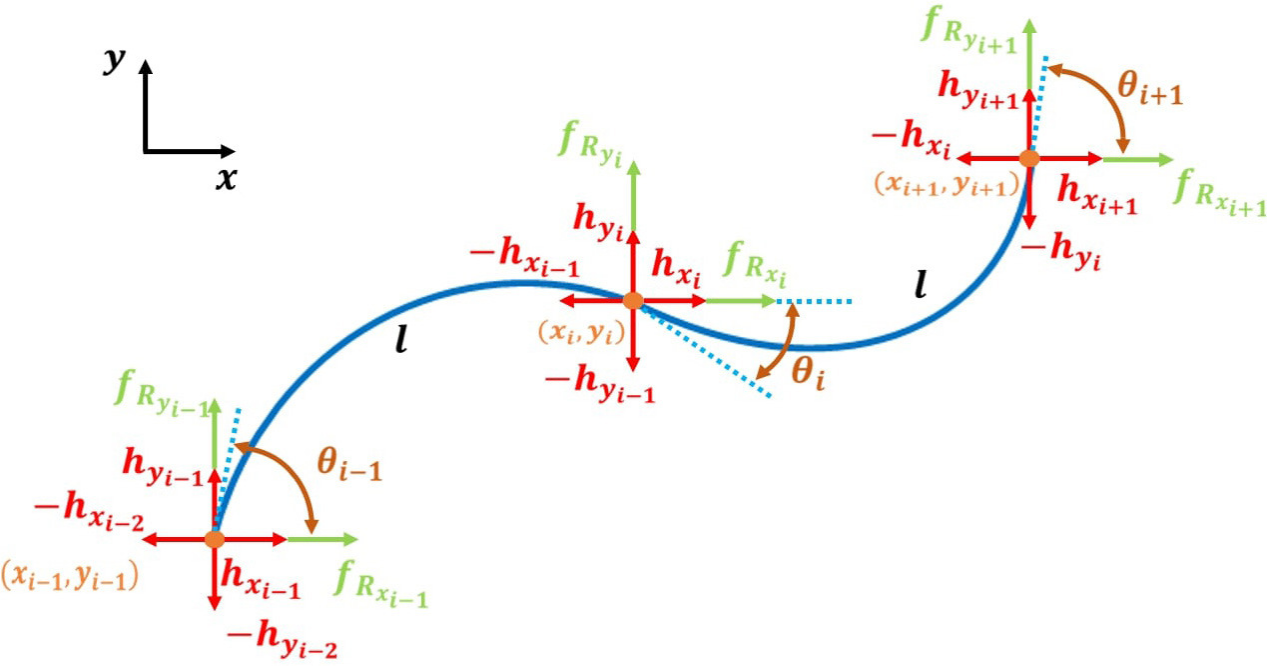
The moment arm of each module of the SRS varies as a function of bending curvature, which follows a second order dynamic response under pressure input. Force and torque balance is maintained under anisotropic friction (*f*_*R*_) and joint constraint forces (*h*) for each node.

In previous work (Luo et al., 2014, 2015a), we presented a mathematical model to predict the dynamic behavior of WPI SRS. We will utilize this model for iterative learning control and motion planning algorithms in a simulation environment, in order to study the proposed algorithms in a controllable manner without the need for repeated experimentation. There are three expressions at the core of our dynamic model. From Figure 3, the force balance equation for each link *i* is written as:

(2) $$\begin{array}{l} \begin{array}{c} m\ddot{X_{i}}=f_{R,x,i}+h_{x,i}-h_{x,i+1},\\ m\ddot{Y_{i}}=f_{R,y,i}+h_{y,i}-h_{y,i+1}. \end{array} \end{array}$$

The torque balance equation for each module *i* is:

(3) $$\begin{array}{l} j\ddot{\theta_{i}}=T_{i}-T_{i-1}+l_{i-1}\left(h_{x,i-1}^{*}-h_{x,i-2}^{*}\right)+\\ \;+l_{i}\left(h_{x,i+1}^{*}-h_{x,i}^{*}\right)+l_{i-1}f_{Rx,i-1}^{*}+l_{i}f_{Rx,i+1}^{*}, \end{array}$$

The dynamic response of the actuator behaves as a second-order system:

(4) $$\begin{array}{l} \kappa =C_{1}e^{\left(-t/\tau_{1}\right)}+C_{2}e^{\left(-t/\tau_{2}\right)}+C_{0}, \end{array}$$

where τ_1_, τ_2_ are time constants and *C*_0_, *C*_1_, and *C*_2_ are constant parameters. Table 1 lists the kinematic and dynamic parameters of the SRS model. The balance of forces and torques at each end of each module is shown in Figure 3.

**Table 1:** Parameters of the SRS dynamic model.

**Symbol**	**Description**
*l*	The constant arc length of soft modules
*m*	Mass of each link
*j*	Moment of inertia of each link
*X, Y*	Centroid global coordinates of each link
*T*	Torque vector (due to pressure input)
κ	The curvature of each module
*f*_*R,x*_, *f*_*R,y*_	Ground friction force at each link
*h*_*x*_, *h*_*y*_	Joint constraint force for each module

## 4. Iterative Learning Control

Under ideal circumstances, a SRS using Equation (1) with ϕ = 0 should travel in a straight line. However, we have observed in our previous work (Luo et al., 2015a) that such a gait will cause the snake to veer off slightly to one direction. This is a result of differences in the behavior of the actuators that make up the snake. These slight differences, resulting from variations in fabrication, air flow rate, and weight distribution between modules result in non-straight trajectories even though all gait parameters are identical. We propose to solve these differences using Iterative Learning Control.

Iterative Learning Control (ILC) is a method for control of periodic systems (Moore and Xu, 2000). The serpentine locomotion of our snake robot is a good example of this, with motion stemming from the repetition of a single gait cycle across modules with a phase difference. Compared with other methods, ILC has minimal computational requirements while maintaining the dynamic behavior of the serpentine gait.

Thus, ILC is an ideal tool to help SRS-4 follow a straight line using only its on-board curvature sensors. The standard formulation of the ILC we use to adjust input parameters is as follows:

(5) $$\begin{array}{l} u_{n}=u_{n-1}+ke_{n-1}, \end{array}$$

where *u*_*n*_ is a control input at iteration *n* (which corresponds to a single period of the sinusoidal curvature wave), and *k* is a control gain. Since the locomotion method of the SRS-4 is a traveling sine wave, the control input *u*_*n*_ represents the PWM duty cycle of the solenoid valves connecting each actuator chamber to the common pressure source. Our current prototype has four modules, hence, eight control inputs. *e*_*n*−1_ is the error between the bending angle amplitude of each module of the previous period and the desired amplitude.

**Figure 4:**
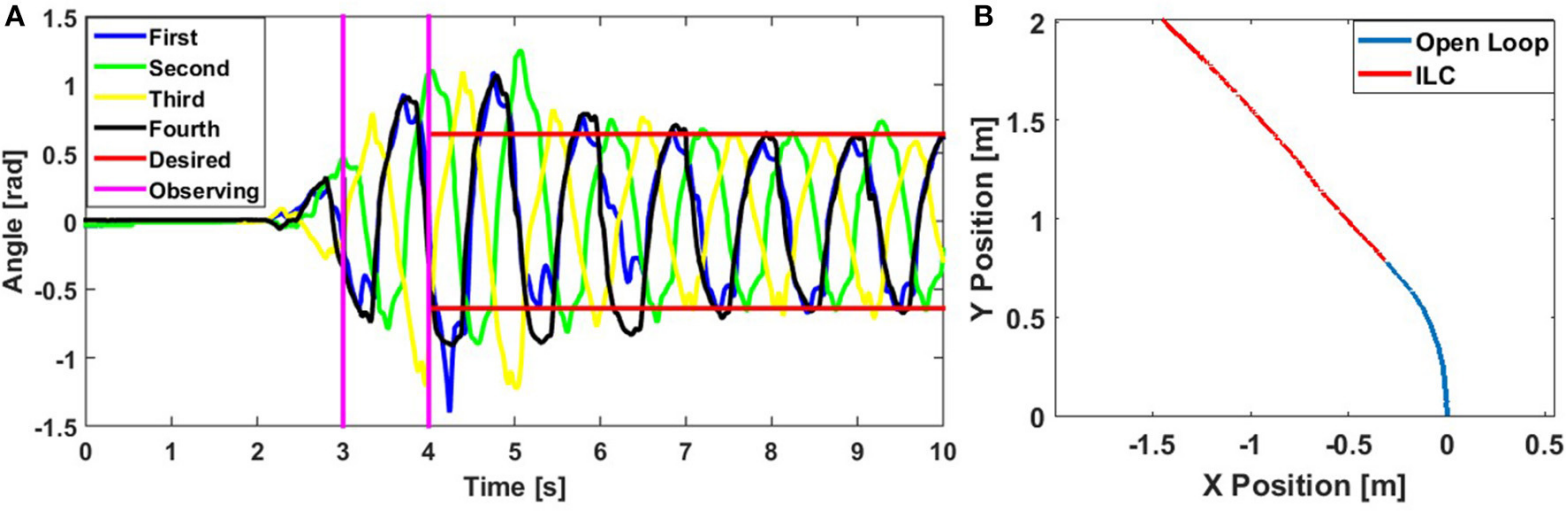
**(A)** Bending angle of each module of the WPI SRS while performing a straight locomotion test controlled by ILC. The blue, green, yellow, and black curves correspond to bending angles of each module from head to tail. The region between the pink vertical lines is when ILC is performing the initial observation process, where the controller records all angle data and pick up the smallest amplitude as the desired angle for the following iterations, as shown as red horizontal lines. **(B)** Centroid of the WPI SRS-4 following a straight line using ILC.

First, we use the basic ILC method of self-learning on the WPI SRS-4. Figure 4A shows bending angle of the four modules during the control process. The gait frequency is 1 Hz, steering offset is 0 with full duty-cycle PWM signal under 19 psi input pressure. We use open loop control for the one and half periods (to the left of the first vertical pink line). The amplitude is increasing during these two cycles as the SRS-4 converges to the steady-state flow rate. The controller starts by recording the bending angles at the beginning of the third period and calculating the positive and negative amplitude of the four modules in this period. Then we choose the smallest measured amplitude as the desired value, ensuring that this amplitude can be reached by all modules.

In the next period, the controller recollects the bending angle of all modules at the beginning of the period, finds the error with respect to the desired amplitude, and passes these errors into the ILC controller to correct the duty cycle of each solenoid valve. At the software level, we can achieve this process on our microcontrollers using time interrupts, which, as only one interruption is required per cycle, results in minimal disruption on gait control. From Figure 4A, we can see that all bending angles are close to the desired angle after ILC takes over (to the right of the last vertical pink line). In this controller, we used a control gain *k* = 0.3.

ILC exhibits a gentle convergence to the desired amplitudes. In the first 2 periods after the ILC kicks in (between 4 and 7 s), many of the bending modules still overshoot the desired amplitude. This is because the air flow rate into the SRS increases for a few seconds after startup, for which the ILC is unable to compensate immediately. This is clearest in the negative bending direction with the first module. After the 3rd cycle, the correction in control input is enough that the valves are no longer saturated, and the actuators behave as desired. Figure 4B and Supplementary Video shows the trajectory of the Centroid of the SRS-4 for this experiment. From the plot, we can see that the use of the ILC allows the robot to travel in a straight line (red portion) even though it starts in a curved path initially (blue portion).

Appendix shows the experimental comparison between ILC and PID controllers, and the result shows ILC has faster speed and less uncertainty at high frequency gait than PID. Similarly, we can also use ILC to maintain a constant curvature steering trajectory. The only difference between this and the straight-line case is that here we choose the positive desired amplitude and negative desired amplitude separately to generate an offset.

## 5. Motion Planning and Trajectory Tracking

Mobile robot motion planning algorithms that use global sensing have been well-established over the last couple of decades (Moore and Xu, 2000; Choset, 2005). Existing work on 2D obstacle avoidance for passive wheeled snake robot motion planning has evolved from these classical mobile robot motion planning algorithms. Early work in this area focused on a kinematic snake motion planning algorithm (Reznik and Lumelsky, 1992). Choset and Henning (1999) created a roadmap using the Generalized Voronoi Graph method, which refines the local paths to take into account the constraints of serpentine locomotion. More recently, R. Liscano's group search for a feasible path using artificial potential fields, then rechecks each local path with modified simulated annealing in order to avoid local minima (Yagnik et al., 2010). Another approach involves linearizing the snake segment rotation into two separate motions and utilizing an existing motion planning algorithm (Liljebäck et al., 2012). Compared to these existing results that provide effective solutions for rigid snake, a soft robotic snake body creates additional challenges that need to be addressed. Having a continuously deformable body, it is difficult to represent the overall shape of the robot and perform collision and distance computations in a reasonable time frame. In addition, the dynamics of the SRS-4 are slower and have a larger effect in the motion response than for rigid snake robots. Its continuously deforming body also means that incorporating the dynamics would be prohibitively time consuming when calculating local paths, and that a linearization method is currently not feasible. Thus, the existing body of work is not directly applicable for the motion planning and trajectory tracking tasks for a soft robotic snake.

**Figure 5:**
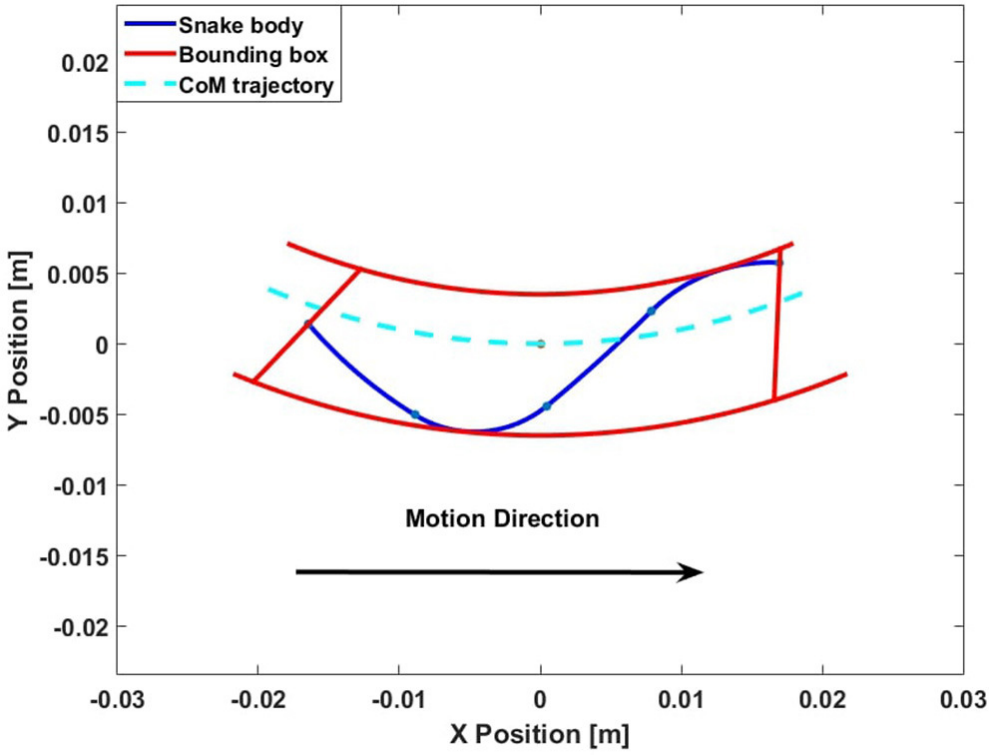
Bounding box definition.

The discrete space A* algorithm is one of the most common and simple tools for mobile robot motion planning in 2D environments because many rigid mobile robots can be represented as a point in the environment. However, discrete space is not realistic for the WPI SRS, because of its minimum turning radius limitations, especially for applications in confined spaces. In this work, we propose the use of an “adaptive bounding box,” as a simple virtual structure that represents space that the WPI SRS occupies, taking into account both its kinematic and dynamic state, considering the area swept by the deformable body during lateral undulation. An example of our adaptive bounding box is depicted in Figure 5, which is constructed simply by translating the Centroid locomotion trajectory laterally and bounding these arcs with line segments on the anterior and posterior of the body.

To calculate the bounding box, we first assume that the WPI SRS Centroid trajectory (the dashed line) is a constant curvature arc. To form the sides of the bounding box, we shift the constant-curvature centroid trajectory in two opposite directions, increasing or decreasing the curvature but keeping the center of curvature the same. These edges are moved outwards until they no longer overlap the edges of the snake. The other two ends of the bounding box are set to be line segments perpendicular to the head and tail angles of the snake. The red curve in Figure 5 shows the bounding box. The adaptability of this shape is crucial for motion planning tasks, such that it is designed to minimally span the area that the locus of body shapes will sweep. Thus, when the body shape changes, the bounding box also changes accordingly, and the locomotion gait parameters have a direct effect on the shape of the adaptive bounding box.

We found that, practically it is beneficial to keep the input pressure and gait frequency constant during locomotion, and thus, the distance from the Centroid trajectory to the left and right boundaries of the adaptive bounding box is a function of the steering offset in the gait algorithm.

The advantage of this approach is a computationally feasible level of abstraction for our soft snake robot to perform motion planning in an obstacle course, despite is relatively complex body deformation. We put this concept into a sampling based motion planning algorithm and bound the problem using the following assumptions:

- The workspace is a flat and continuous surface.
- The linear velocity of SRS-4 is constant regardless of steering.
- All the environmental obstacles can be represented as circles, while the boundaries of the environment are impassible walls as well.
- The Centroid trajectory of SRS-4 is a constant curvature arc during the sampling time.
- WPI SRS-4 is a non-holonomic system with its turning angle bounded by (−α_*max*_, α_*max*_) over the sampling time.

This motion planning algorithm operates in continuous space because the shape of WPI SRS-4 cannot be ignored with respect to the environment. For simplicity, our implementation uses a constant sampling time. Since SRS-4 is a non-holonomic system without differential drive capabilities, it has a finite minimum turning radius. As the experimental results show in Luo et al. (2015a), the Centroid trajectory of the WPI SRS is curvature-bounded and the linear velocity can be treated as constant at different turning offsets (also shown in **Table 3**).

Our motion planning algorithm for soft robotic snake locomotion is based on the Bidirectional A* algorithm. We uniformly divide the turning angle range of WPI SRS-4 [−α_*max*_, α_*max*_] into *N* different curvatures. These *N* curvatures will be each held for a fixed time step (sampling time), resulting in *N* discrete trajectories, or motion primitives, available for the planner at each step. Thus, each parent node will have *N* child nodes. Because we know the turning angle α ∈ (−α_*max*_, α_*max*_) and the initial Centroid motion direction θ, and since the linear velocity *v* and the sampling time Δ*t* are treated constant for node *n*, we can determine the adaptive bounding box shape between nodes *n* and *n* + 1. Given a current Centroid pose: *P*_*x*_(*n*), *P*_*y*_(*n*), θ(*n*) as the position and orientation of the node *n* (see Table 2 for parameter definitions), the Centroid position and orientation of each child node *n* + 1 can be calculated by:

(6) $$\begin{array}{l} P_{x}\left(n+1\right)=r\left(sin\left(\theta \left(n\right)\right)-sin\left(\theta \left(n+1\right)\right)\right)+P_{x}\left(n\right),\\ P_{y}\left(n+1\right)=r\left(cos\left(\theta \left(n+1\right)\right)-cos\left(\theta \left(n\right)\right)\right)+P_{y}\left(n\right),\\ \;\theta \left(n+1\right)=\theta \left(n\right)+\alpha ,\\ \;r=\frac{v\Delta t}{\alpha }. \end{array}$$

**Figure 6:**
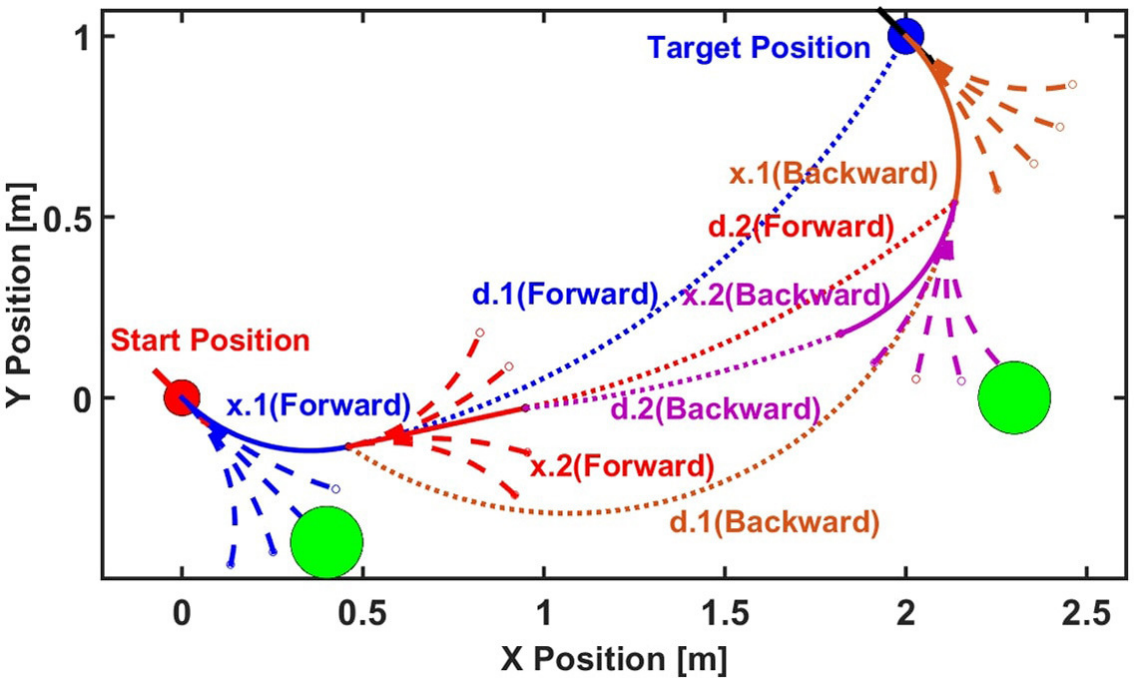
A diagram of the bidirectional A* motion planning algorithm discussed in this paper. The A* algorithm is executed both from the start to target and from the target to start at the same time, increasing the computational efficiency.

Figure 6 shows a simple example of the motion planning algorithm with: *N* = 5, *v* = 1 m/s, Δ*t* = 0.5 s, start pose: (0, 0, −π/4), and target pose: (2, 1, −π/4). As in the regular A* algorithm, we define two scores as:

- *g-score*: If the robot follows a straighter path (with smaller curvature), the g-score will be lower. By limiting the algorithm to sample an odd number of trajectories (i.e., *N* is an odd number), all paths are symmetric with respect to the path *i* = (*N* + 1)/2, which is a straight line. Formally, we define g-score as:
  $$g=\{\begin{array}{ll} i & \;\text{if }i<\left(N+1\right)/2\\ \left|i-N+1\right| & \;\text{if }i>\left(N+1\right)/2\\ 0 & \;\text{if }i=\left(N+1\right)/2 \end{array}$$
- *h-score*: If the arc distance between the current node and the target is smaller, the h-score will be lower. If there is collision with the bounding box and obstacles or borders, the h-score will be a very large number. Assuming *P*_*n*_ is the current node.
  $$h=\{\begin{array}{ll} 10000 & \;\text{if collision}\\ \text{ArcDistance}\left(P_{n},P_{Target}\right) & \;\text{otherwise} \end{array}$$

**Table 2:** Parameters of the motion planning algorithm.

**Symbol**	**Description**
α	Turning angle between nodes
*r*	Arc length of each motion trajectory
*P*_*x*_, *P*_*y*_	Centroid position
θ	Centroid angle
*v*	Centroid Linear velocity
Δ*t*	Sampling time step

The algorithm in this example operates as follows:

1. We start by calculating *N* = 5 child nodes of the start pose, the trajectory of each represented by a blue dashed line. We calculate the h-score of each child node and also check for overlap between obstacles, the edges of environment and the corresponding adaptive bounding box. The bounding box is made of two arcs and two lines, so collision checking is done by checking the radial distance between the center of curvature positions and simple linear projection on the line segments. We then calculate that *x*.1(*Forward*) is the closest node to the target point without collision. The blue solid line shows the Centroid trajectory from the start point to the node *x*.1(*Forward*). The blue dotted line shows the arc distance between the target point to the node *x*.1(*Forward*).
2. As the algorithm is bidirectional, we next calculate the 5 child nodes of the target position backwards. Then, we calculate the arc distance between the children nodes and the node *x*.1(*Forward*). The node *x*.1(*Backward*) has the smallest arc distance respect to *x*.1(*Forward*) without collision based on the score. The brown solid line shows the Centroid trajectory to the target point from the node *x*.1(*Backward*). The brown dotted curve shows the arc distance between *x*.1(*Forward*) and *x*.1(*Backward*).
3. We next iterate in the forward direction, calculating the 5 child nodes of *x*.1(*Forward*) and observe that *x*.2(*Forward*) is the closest node after calculating the arc distance with respect to *x*.1(*Backward*) without collision. We repeat this process, calculating the child nodes of *x*.1(*Backward*) and find that *x*.2(*Backward*) is the closest node after calculating the arc distance to *x*.2(*Forward*) without collision.
4. We keep growing the tree from both directions until one of the following happens: (1) The arc distance between the two newest nodes from the two directions is lower than a threshold and the difference between the orientations is close to π (forward and backward paths are along approximately opposite directions). In this case, we can recover the whole path based on the forward and backward nodes. (2) A time-out occurs, indicating that the algorithm cannot find a continuous path with the given parameters.

**Figure 7:**
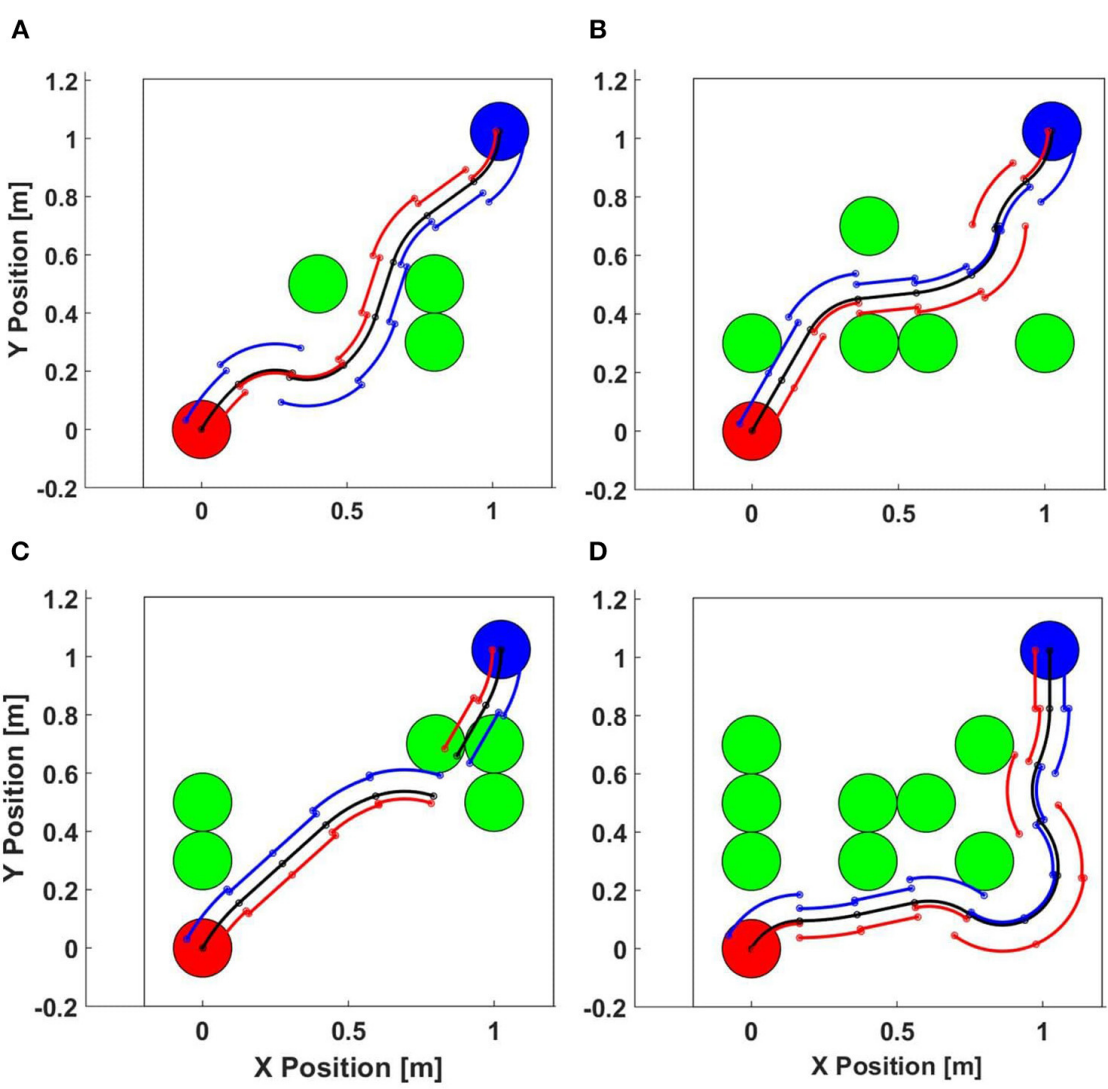
Motion planning algorithm simulation. **(A)** 3 obstacles (Succeeded), **(B)** 5 obstacles (Succeeded), **(C)** 5 obstacles (failed), **(D)** 8 obstacles (Succeeded).

In order to test the performance of this motion planning algorithm, we created a custom simulation environment, as shown in Figure 7. In this example, the start pose was (0, 0, π/4) while the target pose was (2, 1, −3π/4) with a constant linear velocity *v* = 200 mm/s and max turning angle α_*max*_ = π/3 with a sampling resolution *N* = 21. The width of the bounding box is fixed at 50 mm and the right and left distances shift linearly with turning angle, up to 10 mm at the maximum turning angle. There are up to 18 obstacles in a 6 × 3 grid, each with a diameter of 200 mm.

**Figure 8:**
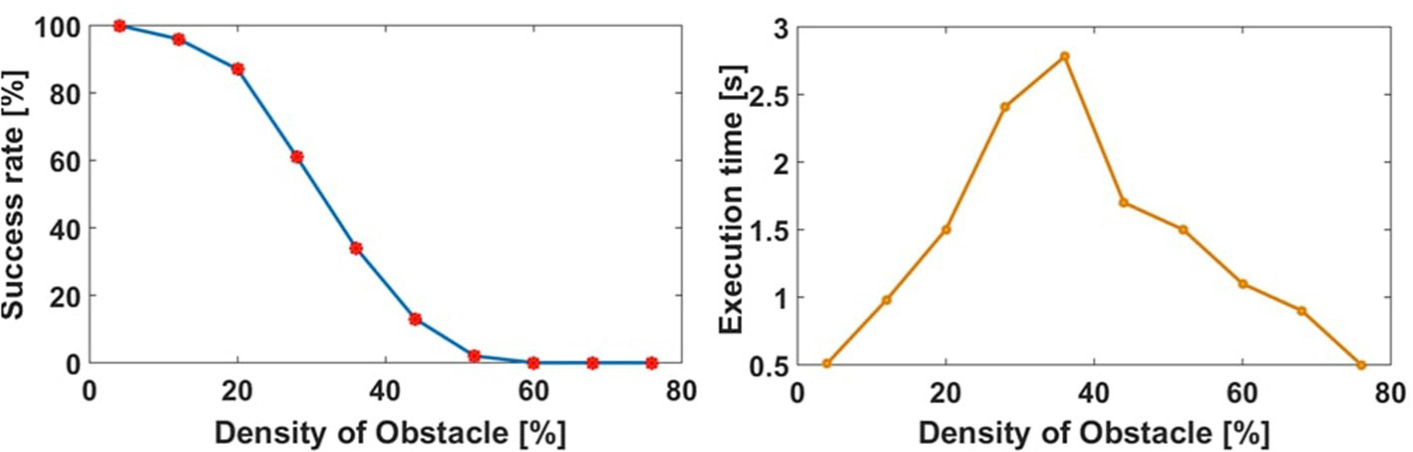
The performance of the motion planning algorithm in the simulated obstacle course **(left)**. The average running time of the motion planning algorithm in the simulated obstacle course **(right)**.

We randomly choose the number of obstacles from only 1 obstacle to all 18. The algorithm ran 100 times for each obstacle density with randomly placed obstacles on an 8-core Intel Core i7 CPU 930 2.80 GHz PC. Figure 8 left figure collates the results of this test. As the obstacle density is increased, the success rate decreases as expected. We note that this test case was designed to study the effectiveness of our motion planning algorithm in relatively tight spaces, with deliberately difficult initial and target orientations pointing toward the obstacle course. Thus ensures that the paths will be close to obstacles and there will be many cases when there are no feasible paths (e.g., Figure 7C). We note that the proposed algorithm is not exhaustive and does not guarantee optimality, but it is computationally efficient.

We recorded the execution time of the algorithm for a given number of obstacles, which can be seen in Figure 8 right figure. This shows that smaller and larger obstacle densities result in faster run times because it is simpler to find a feasible path or terminate. Near the middle obstacle density values, especially when there is no feasible path, the algorithm needs more time to search and exhaust all options.

### 5.1. Trajectory Tracking Control

After we generate a desired path using the previously discussed motion planning algorithm, we need to control the WPI SRS-4 to follow the path. Each node on the path is the desired pose of the robot at each fixed time step. As in a traditional rigid snake robot (Liljebäck et al., 2012), we adjust the locomotion steering offset using the error between the current orientation and the slope of a line between the current Centroid position and the desired node position. We also set a proximity threshold, which indicate that the SRS-4 is close enough to the desired point in continuous space. Therefore, the robot travels toward the first desired position as a waypoint, then the second, and so on until it is close enough to the final desired location. The motion plan ensures that the trajectories can be executed by the robot, and the final pose is achieved as a direct consequence of following the waypoints along the way. The trajectory following control is formulated as:

(7) $$\begin{array}{l} \phi =atan2\left(\frac{Y_{waypoint}-P_{y}}{X_{waypoint}-P_{x}}\right)-\theta , \end{array}$$

where ϕ is the locomotion steering offset, (*X*_*waypoint*_, *Y*_*waypoint*_) are the waypoint positions from the planned path, (*P*_*x*_, *P*_*y*_) is the Centroid position of the robot, and θ is the orientation angle of the robot.

### 5.2. Motion Planning and Trajectory Tracking Control Simulation

**Figure 9:**
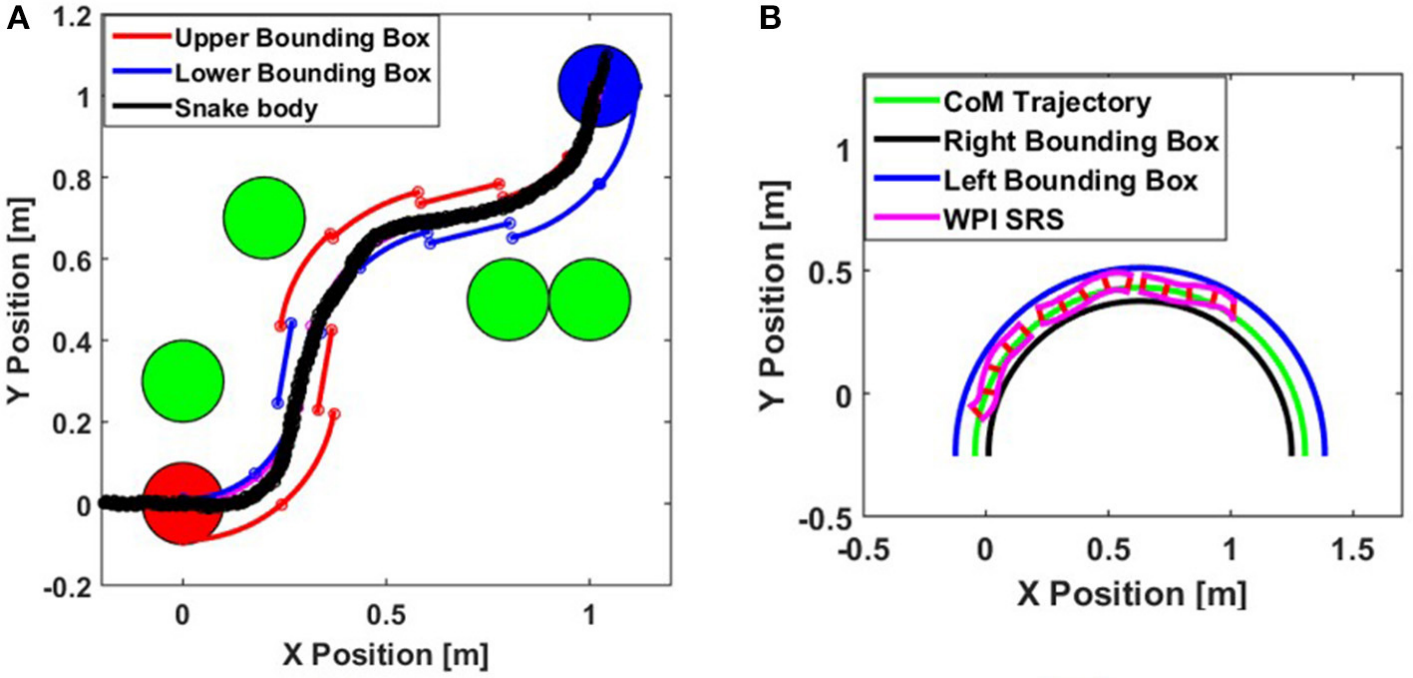
**(A)** Motion planning and trajectory following simulation of WPI SRS. **(B)** Experimental characterization of the adaptive bounding box parameters.

We simulated our soft robotic snake in an obstacle course test environment in order to test the motion planning and trajectory tracking algorithms. First, we plan a path based on the environment and the WPI SRS kinematic and dynamic information, and subsequently control the locomotion steering offset parameter of the robot to follow this path. For these simulations, we set the linear speed of the SRS to be 200 mm/s and the width of the bounding box to be 50 mm (shifted in the lateral direction to adapt to different steering offset values). We picked a bounding box width to be a little larger than the locus of body shapes of the robot, which allows for additional safety. We used a sample time Δ*t* = 1 s, with 4 randomly positioned obstacles in the environment. Figure 9A shows that the robot body remains inside the bounding box as it follows the entire trajectory and that the WPI SRS accurately follows the desired trajectory.

### 5.3. Experimental Verification

We verified the soft snake motion planning algorithm though physical experimentation. The procedure as follows: First, we experimentally determined the control properties of the 4th generation WPI SRS: Linear velocity, bounding box size and trajectory curvature bounds. Next, we plan a path for the WPI SRS-4 using the previously-discussed motion planning algorithm and finally, we control the SRS-4 along this pre-defined path.

We ran the system using different steering offsets ϕ, from −0.3 to 0.3, using the ILC. With a steering offset of 0, the SRS-4 will follow a straight line and the desired bending angle amplitude the ILC uses for each direction of each module is 0.7 rad. When the steering offset is between 0 and 0.3, the robot will turn in one direction.

When using the ILC for this behavior, we keep the desired bending angle amplitude for the large bending direction to be 0.7 rad and reduce the desired amplitude for the smaller bending direction to be between 0.4 to 0.7 rad, which is linearly interpolated from the steering offset range (0.3 to 0). When the steering offset is between −0.3 and 0, the WPI SRS-4 will turn in the opposite direction and the desired angle for two directions of bending are flipped. We record the position and orientation data of the SRS-4 using an OptiTrack motion capture system. We determined the adaptive bounding box parameters by characterizing the body shapes for different offset values and identical input pressure and frequency values. Figure 9B shows the bounding box from a representative experimental result with undulation frequency: 1.5 Hz and steering offset: 0.3.

We recorded the position of the entire body of the WPI SRS-4 during its movement, and calculated the radius of curvature of its trajectory. To calculate the size of the corresponding bounding box, we calculated the radii that bound the positions of the SRS on the inside and the outside of its trajectory. Table 3 collects the adaptive bounding box parameters extracted from this experimentation, while Table 4 shows the values of the parameters we used for the motion planning algorithm. As a safety factor, we used an approximately 50% wider bounding box for motion planning than the largest width calculated experimentally. The linear velocity of the WPI SRS is assumed constant around 0.12 m/s for planning purposes. The turning radius is not symmetric with respect to the steering offset, and exhibits some bias (i.e., moving in a circular path of a large diameter for zero offset) due to manufacturing and assembly variations. We choose the smallest turning angle (for each 1-s time step) measured from all experiments as an achievable bound on trajectory curvatures, using a maximum turning rate of 9 degrees-per-second for motion planning.

**Table 3:** Parameters extracted from experimental calibration data for the adaptive bounding box.

**Offset**	**Linear velocity [m/s]**	**Left bounding box [m]**	**Right bounding box [m]**	**Radius of curvature [m]**
−0.3	0.1471	−0.09	0.055	−0.6727
−0.2	0.1489	−0.09	0.06	−0.8445
−0.1	0.1696	−0.11	0.08	−1.7707
0	0.1345	−0.1	0.1	4.467
0.1	0.1524	−0.08	0.1	1.8152
0.2	0.1082	−0.07	0.1	1.2532
0.3	0.1216	−0.06	0.09	0.7856

**Table 4:** Parameter values used in the motion planning algorithm.

Max turning angle	9^*o*^
Linear velocity	0.12 m/s
Bounding box size	0.25 m
Max shift	0.05 m

We experimentally verified the performance of the motion planning and trajectory following algorithms in obstacle navigation scenarios. First, the tracking system sends the position of the obstacles and robot's centroid to the computer though wireless communication. The computer defines a planning path using this information and the chosen parameters values. Then the robot follows each waypoint of the path by adjusting its steering offset parameter. During this motion, the ILC controller uses the onboard curvature sensors to keep module bending amplitudes to remain consistent which ensures that the trajectory of the robot follows a desired radius of curvature arc, to follow the path more precisely.

**Figure 10:**
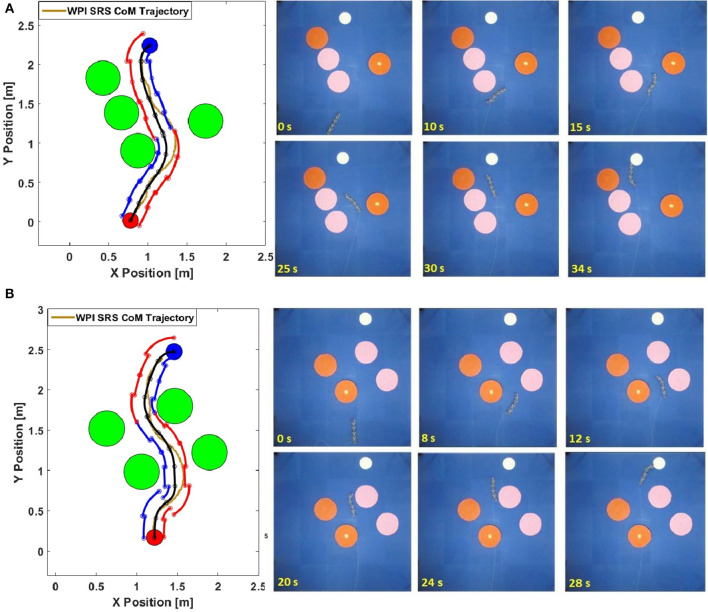
**(A)** Experimental results of the first test scenario (Target angle is 60^*o*^). Top figure shows WPI SRS-4 body overlaid on the planned path and bounding box at different times during the experiment. Panels show snapshots of the experiment. **(B)** Experimental results of the second test scenario (Target angle is 0^*o*^). Top figure shows WPI SRS-4 body overlaid on the planned path and bounding box at different times during the experiment. Panels show snapshots of the experiment.

Results of our experiments are shown in Figure 10. In this figure, we present the planned path and a locus of the soft body of the WPI SRS-4 as it goes through the course. Figure 10A and Supplementary Video shows a simple scenario, where the target body orientation is 60° (from horizontal). From the plot, the desired path shows the SRS-4 may reach the target by navigating the gap between these obstacles. We can see that the snake robot has trouble following some of the tight curves dictated by the planner and exhibiting overshoot, despite the fact that these curves were calibrated to be achievable by the robot at steady state.

This effect is more prominent in the second validation scenario (Figure 10B), which positions the obstacles closer together to force an S-like path. The desired final orientation is also reduced to 0°. These changes make the tasks of motion planning and trajectory tracking more difficult to focus on the effect of transitions between planned path sections. While SRS-4 can find a feasible path and follow it to the target pose, it exhibits overshoots and oscillations around the desired path, although it remains within the planned boundaries during locomotion.

We believe two factors cause this inconsistency between calibration behavior and actual behavior: first, the speed of wireless communication between the snake and the base computer is slow, resulting in delays in the onset of commands. The trajectory tracking algorithm runs at around 1,000 Hz on the base computer and the frequency of the local ILC control is around 60 Hz because of the limited bandwidth of the binary solenoid valves. Second, and more significantly, the transition between two “distant” locomotion modes suffers from dynamic effects that aren't taken into account for planning purposes. When there is a large change in the steering offset values between path sections, the snake cannot immediately change its behavior, since the trajectory evolves through dynamic changes among bending angles of modules. This in turn causes a delay in converging to the next steering offset path, and hence a non-negligible overshoot. However, we see that given a reasonable distance between nodes (or time step), the SRS-4 will converge to the desired pose after several nodes. From this figure, we observe that the robot is able to re-acquire the planned trajectory after making a few oscillations around it due to the large transition near the beginning of the path.

## 6. Conclusion and Future Work

In this paper, we proposed the fourth generation of WPI Soft Robotic Snake (WPI SRS-4) which is a modular soft robotic system. Each soft bending module has its own integrated local controller, solenoid valves, and curvature sensor. Together, these modules can be controlled using a master controller using a I2C serial network, creating an autonomous mobile soft robot. To improve the reliability of path following, we implemented iterative learning control (ILC) using on-board curvature sensors. In addition, we designed an adaptive bounding box motion planning algorithm, which is able to help WPI SRS find the path to navigate obstacles using curvature bounded path sections. This algorithm combines motion primitives with a simplified kinematic footprint of the WPI SRS, allowing it to simply plan achievable paths for this complex soft snake robot. We created a method for the SRS-4 to follow these predetermined paths, and experimentally analyzed their performance.

One limitation of this work is that it does not consider the dynamic behavior of transitions between different motion primitives characterized at steady-state. The snake can not instantaneously switch between locomotion modes, and large jumps can result in a delayed response, and therefore overshoots and oscillations in the robot trajectory. In order to address this problem, future work will increase the speed of wireless communication, study continuous changes in steering offsets to avoid rapid switching between two locomotion modes, and incorporate dynamic transition effects into the motion planner.

While outside the scope of this work, we acknowledge limitations to the use of pneumatics as a driver of soft, mobile robots. Currently there are no air compressors available that are both powerful enough to drive the SRS and small enough to be carried on board without drastically reducing speed, something we encountered in Luo et al. (2015a). The goal of soft mobile snake robots will not be achieved until this problem is solved, though, it should be noted that rigid snake robots also suffer from power consumption problems and often require a tether.

One additional direction we would like to investigate is the use of the WPI SRS for manipulation. The compliant continuum structure of this robot makes it useful for wrapping around objects and moving them. This would particularly be a challenge because most current work in this field assumes that a manipulator has a static base, or mobile bases that have certain simply modeled kinematic behaviors. We would also like to examine the number of modules used in the WPI SRS and study its scalability. Increasing the number of modules may improve performance on certain tasks, but this may also increase computation time and energy costs. Certain tasks may have an ideal number of modules. For example, a manipulation task may need more modules for redundancy, while a locomotion task may need fewer.

It has been observed that in rocky or cluttered environments (Astley and Jayne, 2009), biological snakes take advantage of obstacle contact in their locomotion, pushing off of the environment to increase efficiency. We would like to investigate techniques to allow the WPI SRS to mimic this behavior in a usable way. For example, this would involve modeling and sensing the state of the SRS without the constant-curvature assumption.

Finally, the current SRS-4 prototype is only able to effectively traverse a 2D surface, making it significantly less capable than its biological counterpart (and some rigid robotic snakes, Yamada and Hirose, 2006; Transeth et al., 2008), which can traverse complex environments with four different locomotion types. We would like to expand each module of the SRS to allow for these more complex motions and locomotion types. We would also like to expand the motion planning and control techniques in this article to these more complex soft robotic snakes, creating 3D bounding boxes, which can factor in the complex curvatures of modules in contact with objects.

## Data Availability Statement

The original contributions presented in the study are included in the article/Supplementary Material, further inquiries can be directed to the corresponding author/s.

## Author Contributions

ML: mechanical design and fabrication, algorithm developing, and testing. ZW and YS: fabrication, low-level control developing, and testing. ES, WT, and FC: mechanical design and fabrication. LG and HY: testing. CO: principal investigator and idea provider. All authors: contributed to the article and approved the submitted version.

## Conflict of Interest

The authors declare that the research was conducted in the absence of any commercial or financial relationships that could be construed as a potential conflict of interest.

## Figures and Tables

**Figure A1:**
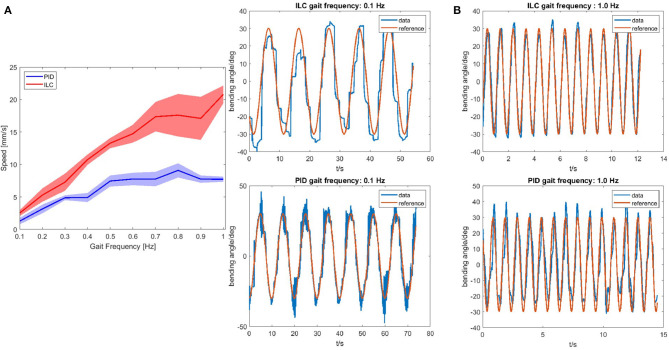
**(A)** Relationship between gait frequency and speed of 2D soft robotic snake under ILC and PID controller. Shade shows the standard deviation of the result. **(B)** Bending angle and reference for one of the four modules on the snake controlled with ILC controller and PID controller tracking sinusoidal waves under the gait frequency of 0.1 and 1.0 Hz.

## A. Appendix

During the Control of our 2D snake robot, the output of the controller is PWM signal for solenoid valves. The highest frequency of PWM signal that the solenoid valves can recognize is 60 Hz, thus the control frequency is limited to a considerably low value. In this section, experiments are conducted to compare the performance of ILC and PID controller under lower control frequency.

A closed-loop proportional-integral-derivative (PID) controller is a simple feedback control algorithm most commonly used in industrial control systems. Thus, we implemented a PID controller as a benchmark to compare with the iterative learning control (ILC) strategy we proposed in this article. Instead of only using the maximum module bending angle, the PID controller tracking sinusoidal wave signals for the bending angle of each snake module by adjusting the PWM duty cycle for each slave controller via I2C to ensure accurate trajectory following. Each controller utilized its own set of control coefficients manually tuned for best performance.

The test is conducted for 10 different gait frequency from 0.1 to 1.0 Hz with step of 0.1 Hz, and the phase offset is $\frac{2\pi }{3}$. For each parameter setting, the experiment is repeated over 5 times to get a significant analysis of the results.

Figure A1A shows the relationship between gait frequency and speed of our soft robotic snake. Generally as the gait frequency increasing, the speed of snake robot is higher. For each gait frequency in the experiment, the snake robot is always faster under ILC controller. Besides, we can observe that when the gait frequency is higher, snake robot becomes slower under PID controller, because when the gait frequency is too high, the sampling number of each cycle will be much smaller, thus the snake modules can not track sinusoidal wave well. As shown in Figure A1B when the frequency is set 1.0 Hz the mean squared error between the sinusoidal wave signal and experimental result is 83.52 *deg*^2^, thus snake robot can not perform well-generated locomotion. However, when the robot snake is controlled with ILC controller under higher gait frequency, the bending angle of each snake robot module fit the sinusoidal wave signal much better with a mean squared error of 24.64 *deg*^2^ even though the ILC controller confirms a square wave signal. Our research before (Onal and Rus, 2013; Luo et al., 2014) shows that for snake robot manufactured with fexible material, under certain gait frequency, square wave signal can result in sinusoidal wave like output because of the damping of soft actuator. As the result, snake modules controlled with ILC controller perform much more smooth sinusoidal waves under higher gait frequency compared to PID controller.

Figure A1B also proves that, when the frequency is lower, PID controller tracks sinusoidal wave much better with a mean squared value of 32.90 *deg*^2^ between singal and experimental result because sampling number during each cycle increases. While ILC controller results in a more square wave like output with a mean squared value of 108.13 *deg*^2^ between singal and experimental result.
